# Exploring the health-seeking journeys of individuals affected by leprosy: Lived experiences in selected urban rehabilitation centers in Ethiopia

**DOI:** 10.1371/journal.pntd.0013938

**Published:** 2026-02-17

**Authors:** Temesgen Kabeta Chala, Esayas Kebede Gudina, Kristina Adorjan, Guenter Froeschl

**Affiliations:** 1 CIHLMU Center for International Health, Ludwig-Maximilians-Universität München, Munich, Germany; 2 Department of Health Policy and Management, Jimma University, Jimma, Oromia, Ethiopia; 3 Department of Internal Medicine, Jimma University, Jimma, Oromia, Ethiopia; 4 Clinical Trial Unit, Jimma University, Oromia, Ethiopia; 5 Institute of Psychiatric Phenomics and Genomics (IPPG), LMU University Hospital Munich, Munich, Germany; 6 Department of Psychiatry and Psychotherapy, University of Bern, Bern, Switzerland; 7 Multidisciplinary Center for Infectious Diseases (MCID), University of Bern, Switzerland; 8 Institute of Infectious Disease and Tropical Medicine, University Hospital (LMU), Munich, Germany; Marie Adelaide Leprosy Centre Pakistan, PAKISTAN

## Abstract

**Background:**

Despite widely available and effective treatment, leprosy remains a major public health issue in Ethiopia. The disease is often misconstrued as a hereditary disease in origin, a curse, or a form of divine punishment for immoral behavior. In this study, we aim to explore how individuals affected by leprosy perceive the disease, recognize its early symptoms, and how these perceptions influence care-seeking behaviors.

**Methods:**

An exploratory qualitative study was conducted at selected leprosy treatment and rehabilitation centers located in Addis Ababa, Shashemene, and Jimma. In-depth interviews (key informant interview (KII)) and focus group discussion (FGD) were conducted. Interviews were transcribed verbatim in local languages, translated into English, and reviewed to identify key themes. Data were coded in MAXQDA 24 using pre-identified themes (perceived causes, stigma, social consequences, care-seeking) and open coding to include emerging themes.

**Results:**

A total of 8 FGDs with 53 participants (25 females; mean age 52) and 11 KIIs (5 females) were conducted. Participants demonstrated limited knowledge of leprosy, often attributing it to supernatural causes or divine will, which delayed care-seeking and led to advanced disease stages. Misconceptions and stigma led to social isolation, discrimination, and inter-generational exclusion, with profound economic, social, and psychological impacts. Misdiagnosis or delayed diagnosis, and provider stigma were among the healthcare-related challenges. Despite this, there were individuals that demonstrated resilience by forming supportive social networks, including community-based associations that fostered mutual aid, inclusion, and dignity.

**Conclusion:**

In Ethiopia, leprosy is still a major public health issue. Affected individuals often suffer social exclusions, psychological distress, and diminished quality life. Stigma from health care providers was also reported. Collaborative efforts between the health system, religious leaders, and leprosy affected individuals and their associations are vital for elimination of the disease.

## Background

Leprosy also known as Hansen’s disease is a chronic infectious disease caused by *Mycobacterium leprae.* It mainly affects the skin, peripheral nerves, mucosa of the upper respiratory tract, and eyes [1]. Leprosy was considered as an incurable disease with supernatural causes, that left visible lesions for life, until 1873, when Norwegian physician Gerhard Armauer Hansen identified the bacterium *Mycobacterium leprae* as the cause of leprosy [2]. Even though the global prevalence has declined due to the introduction of antibiotic treatment, improved living conditions and measures of case detection, later complemented by the widespread use of multidrug therapy (MDT), the disease remains a significant public health concern particularly in resource limited settings [3].

The World Health Organization (WHO) reports that over 200,000 new cases occur each year, with countries in the Southeast Asian and African regions having the highest rates of new cases [4]. In Ethiopia, leprosy remains an endemic disease, especially in areas with limited access to healthcare. The country continues to report thousands of new leprosy cases each year. A study conducted among close contacts of leprosy affected individuals in Ethiopia identified 15 individuals for leprosy out of 16107 (9.3 per 10,000) [5]. These cases were not found through passive case detection methods. This suggests there are likely many leprosy cases in the country that remain undetected. With the existing data, the country ranks 7th globally and 3rd in the African region in reporting new child leprosy cases annually, with the majority of the cases having developed grade 2 disability (G2D) by the time of diagnosis [6].

The biological, psychological, and socioeconomic effects of leprosy are substantial, both for the individual as well as for the community [7]. Untreated leprosy can lead to loss of sensation, muscle weakness, and paralysis. It can also result in disabilities and visible deformities, including clawed hands, facial disfigurement, and blindness. Type 1 and type 2 leprosy immune reactions cause symptoms such as pain, fever, and swelling. Sensory loss can result in unnoticed wounds and ulcers, which can result in chronic infections [8]. Similarly, stigma and discrimination, which can lead to social isolation and mental health issues such as depression, anxiety, low self-esteem, and suicidal thoughts, are among the main leprosy’s psychological impacts. Loss of income is another challenge of leprosy, which can be the result of disability and discrimination. Besides, although MDT is free, patients frequently need to pay out-of-pocket for additional medical remedies such as corrective surgeries. The socio-economic effect of leprosy also impacts the caregivers because they stop working to provide support to the patients [9].

Early diagnosis is crucial for the effective management of leprosy. Detecting the cases early helps not only in avoiding complications but also contributes to breaking the chain of transmission. This early detection of leprosy involves recognition of symptoms such as skin patches with loss of sensation, thickened peripheral nerves, and muscle weakness [10].

However, stigma and misconceptions about leprosy are one of the major barriers to timely treatment. As a result, leprosy remains one of the most stigmatized and disabling infectious diseases, leading to avoidable disabilities, psychosocial distress, and economic hardship, pushing affected individuals and their families deeper into poverty, and exposing the affected individuals to social stigmatization and marginalization [11]. Although medical science has advanced, still now many members of affected communities consider leprosy as a curse or a manifestation of evil spirits rather than a bacterial infection [12]. To develop a practical and acceptable intervention strategy, it is essential to understand the perceptions, beliefs, and lived experiences from the affected individuals. The main aims of this study are to explore perceptions of leprosy-affected individuals with a focus on the recognition of clinical manifestations, and how these perceptions influenced their health-seeking behavior.

## Methods and materials

### Ethical consideration

Ethical clearance for this study was obtained from Jimma University, Institute of Health Institutional Review Board, Ethiopia, reference number (JUIH/IRB/022/24) and CIHLMU Center for International Health, Ludwig-Maximilians-Universität München, Munich, Germany, Opinion No. 24–0083. A letter of support was written from Jimma University and the Oromia Health Bureau to the specific study sites. The data collection was initiated after informed consent was obtained. For study participants who were unable to write, oral informed consent was obtained in the presence of an impartial witness. The witness testified to the consent process and signed the form on behalf of the participant to document their voluntary participation. Participants were informed of their right to interrupt the study at any time. Generally, voluntary participation, anonymity, confidentiality, and privacy were ensured throughout the interview process.

### Setting and period

The study was conducted at selected leprosy rehabilitation sites in Ethiopia. Located in the Horn of Africa, Ethiopia is the continent’s second-most populous nation, with an estimated population of over 123 million. The country has a longstanding history of addressing leprosy and is home to several specialized centers dedicated to the diagnosis, treatment, and rehabilitation of individuals affected by the disease. There are six primary leprosy care centers across the country. This includes Relief Center Bisidimo, Dessie Boru Meda, Shashemene/Kuyera, Gambo, Bushulo, and All-Africa Leprosy Tuberculosis and Rehabilitation Training Center (ALERT).

In addition to these government-supported facilities, leprosy care is also provided by institutions established through partnerships with faith-based and non-governmental organizations. Notably, the Catholic Church and the Ethiopian National Association of People Affected by Leprosy (ENAPAL), in collaboration with the German Leprosy and TB Relief Association (GLRA), contribute significantly to service delivery, advocacy, and community-based rehabilitation centers like Jimma.

In this study we selected three sites form different geographic locations. This includes: (a)ALERT, which is located in Addis Ababa and is the largest leprosy treatment center, operating since 1934 and recognized by the WHO as a reference training center on leprosy health care in the country. (b) Shashemene Rehabilitation Center, founded in 1952, which is about 250 km to the east of Addis Ababa. The facility was initially known by the name “Kuyera”, a derogatory term which means ignored, rejected, put aside, or non-valued people. (c) Jimma Leprosy Rehabilitation Center has housed and provided occupational opportunities to leprosy-affected residents since 1974. This was established by faith-based charities and been recognized by on Ethiopian National Association of Persons Affected by Leprosy (ENAPAL). The study was conducted from 16 April 2024–20 November 2024.

### Study design

An exploratory qualitative approach was employed, using KIIs and FGDs. This design allowed to explore and obtain rich and in-depth information from the participants’ lived experiences and perspective on the phenomena under investigation. We adapted the definition of lived experience from Guerrero et al. [13], which refers to individuals who are directly affected by social, health, public health, or related issues, as well as the strategies used to address them.

### Study population

Information about the study was disseminated to the community within and outside the rehabilitation centers through telephone calls and word of mouth, using local languages. Individuals residing outside the rehabilitation centers and those who could not be reached by phone were contacted through house-to-house visits. This was feasible as the people affected by leprosy frequently reside in the vicinity around the rehabilitation areas. As this study was conducted in selected leprosy rehabilitation centers, we identified rehabilitation center coordinators, who functioned as “gatekeepers”. The gatekeepers were in charge to check inclusion criteria in their patient groups as they were consecutively appearing at the centers, and to invite the selected candidates to the KII and FGDs.

Participants were eligible if they had been diagnosed with leprosy at any time, were attending to one of the included rehabilitation centers for at least six months at the time of data collection, were 18 years of age or older, able to hear and speak and were willing to participate. Only individuals that explicitly consented to participate and signed an informed consent (directly or via a testimony) were included. Eligible individuals were invited to attend their respective rehabilitation centers on specified appointments.

To maintain homogeneity, separate interviews were held based on gender and the presence of visible deformities. Initially, we scheduled ten KIIs and six FGDs (two mixed-gender, two female-only, and two male-only). Differentiating criteria for inclusion in the FGDs included gender, disease stage, and community roles. For the KIIs, participants were selected if they were known for their extended living experience with leprosy in consultation with gatekeepers and rehabilitation coordinators.

Data saturation was monitored through continuous review and comparison of interviews, and it was considered reached when no new insights emerged, and responses began to repeat. Although data saturation was reached for ALERT and Shashemene sites, the emergence of new insights from Jimma warranted conducting additionally 2 FGDs and 1 KII, bringing the total number of interviews to 8 FGDs and 11 KIIs.

A total of 53 informants (6–9 per group) participated in the 8 FGDs with the following criteria:Two mixed-gender FGDs were organized at the ALERT site: one involving participant without visible deformities and the other involving participants with visible deformities. A total of four FGDs were conducted at the Jimma site, including a mixed-gender group with visible deformities, a mixed-gender group without visible deformities, a female-only group without visible deformities, and a male-only group without visible deformities. At the Shashemene site, two FGDs were held with male-only and female-only groups, both comprising participants without visible deformities.

### Data collection method

The semi-structured interview guide was developed by adapting the data collection approach described in the previous work of Ebesuno et al [14]. The first guide was prepared in the English language and then translated into local languages (Afaan Oromo and Amharic). The interview guide, pilot tested in Jimma University Medical Center with individuals presenting for tuberculosis management, was semi-structured in order to maintain consistency between the two trained interviewers. The piloting aimed to assess the clarity and estimate the time management for interviews. The results of the piloting were used to refine the interview guide and were not included in the final analysis. In addition, we utilized a structured questionnaire to collect information about socio-demographic characteristics (i.e., age, gender, educational status, and occupation) while using open-ended questions focused on individuals’ lived experiences regarding leprosy and its social impacts from the time of diagnosis to present. Probing questions were aimed at deepening the understanding of how individuals experienced, perceived, and navigated their illness and care process. Interviews were audio-recorded after obtaining informed consent.

We also utilized field notes to complement audio recordings and ensure that non-verbal, contextual, and environmental information is captured. Data was collected by two Master’s students from the School of Nursing and Public Health at Jimma University. Two days of training were given for the data collectors on the interview guide when conducting an in-depth interview. Interviews were held in leprosy rehabilitation centers of the selected sites, where privacy and low ambient noise for recording were optimal. These discussions were conducted in a circular seating pattern where participants engaged face-to-face, which helped the moderators involve all the respondents. We assigned each participant in the FGDs a unique identifier code.

Participants were instructed to refer to themselves by their code when speaking. This helped preserve anonymity during discussions and facilitated subsequent data analysis. Moreover, moderators used these codes to direct questions or encourage participation from specific participants, thereby enhancing engagement while maintaining confidentiality. The time of the interview and discussion was scheduled based on mutual agreement with the study participants.

### Trustworthiness

Methodological rigor of the study was ensured based on the framework of trustworthiness for qualitative studies by Rosenstock IM [15], which we employed the concept for this study. Trust-building with participants, introductions by local coordinators (gatekeepers), active participation, using participants’ own words, and post-data-collection reflection sessions with the data collectors and field supervisors all contributed to credibility. In addition, reliability was preserved through routine comparison of transcripts to audio files, asking clarifying questions, use of probing questions. Furthermore, external validity and comparability were improved using a variety of data collection techniques, such as KIIs with individuals both with and without visible advanced stages of disease, and FGDs stratified by gender, as well as by including diverse and representative participants from various settings.

### Data management and analysis

All transcripts and audio recordings were reviewed, securely stored, and assigned file names (indicating interview type, study participant, study site, and year of interview). The files were saved on a personal computer and backup copies saved on a password secured data repository of the Leibniz-Rechenzentrum of the Bavarian Academy of Sciences. The audio recordings were transcribed verbatim in the local source language (Afaan Oromo and Amharic) before being translated into English for analysis. We conducted multiple readings of the transcripts and field notes to identify key themes, which were then coded using MAXQDA 24. We employed a hybrid coding approach, combining both deductive and inductive strategies. The analysis was guided by a mix of pre-identified themes, such as perceived causes of leprosy, stigma, social consequences, and care-seeking pathways, along with open coding to capture new insights.

The findings were organized into themes, sub-themes, and sub-categories, supported by representative quotes. The analysis provided a detailed understanding of how individuals affected by leprosy viewed the disease, recognized early symptoms, navigated care pathways, and dealt with stigma and social exclusion. The codebook was generated using MAXQDA 24 and has been included in S1 File.

In addition, many words and expressions in local languages were used throughout this manuscript. To enhance clarity and cultural understanding, we developed a glossary explaining these terms, which was provided in S2 File).

## Results

A total of 8 FGDs with a total of 53 participants, and 11 KIIs were conducted. The participants in the FGDs were 25 females and 28 males, with a mean age of 52 (SD = 16) years (ranging from 18 to 79). Among these participants, 31 (58.5%) had no formal education, 19 (35.8%) had a primary school education, and 3 (5.7%) had a secondary school education. Regarding occupation, 46 were unemployed, while the remaining were farmers, a guard, a daily laborer, or a government employee. The average duration of the FGDs was 81 minutes (62–118 minutes).

**Fig 1:**
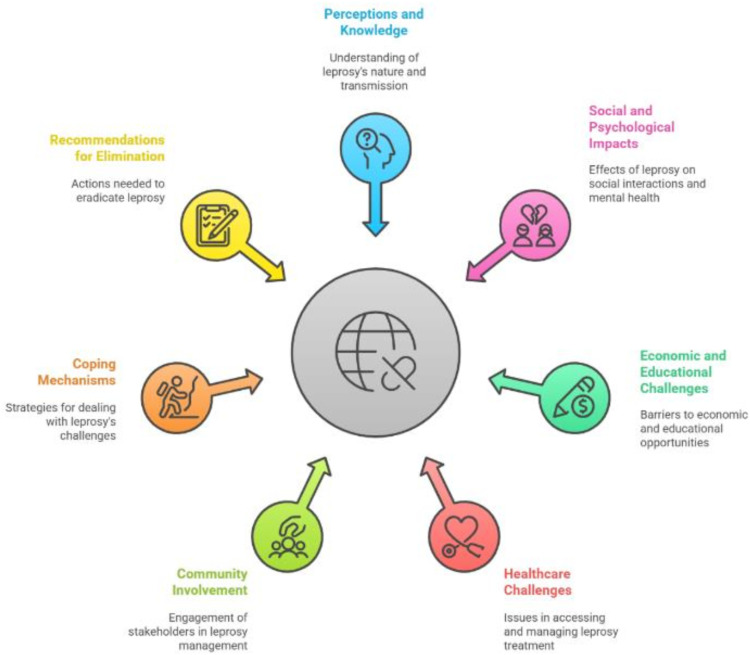
Main themes and sub-themes identified in the analysis (created using Napkin, a visual AI tool for transforming text into info-graphics) [16].

For the KIIs, 5 of the 11 informants were female and 6 males. Three of the key informants held a university degree or higher, two had a primary school education, and six had no formal education. The average interview duration for KII was 44 minutes. This study identified key themes such as: perceptions and knowledge of leprosy; deformities and disfigurement; perceived causes and modes of transmission; misconceptions and awareness; socio -economic and psychological impacts; vulnerability, leprosy management and healthcare related challenges; community and stakeholder involvement; treatment efficacy and the importance of timely care; resilience among affected individuals; and recommendations for leprosy elimination (Fig 1).

### Perceptions and knowledge of leprosy

#### Subjective and objective manifestations of leprosy.

Common initial symptoms mentioned by participants included pale skin patches, pea-sized bumps resembling warts, and a tingling or cold sensation in the affected areas. As the disease progresses, symptoms like dizziness, burning sensations, itching, fatigue, loss of sensation, difficulty grasping objects, and loss of sensation resulting in unnoticed injuries and wounds were reported. A key informant described as: “*I do not feel pain in my hand or toes, so I get burned by fire or cut by sharp objects without realizing it. Eventually these led to development of ulcers and finally result in amputation of extremities” (A 60 years old male, KII 5, 2024*).

Another informant described as: *“At the beginning, I felt a tingling sensation and coldness in parts of my body. Then white spots appeared on my face, hands, and legs. My hands became weak and I couldn’t even hold food to eat” (A 75 years old male, FGD 5, P4, 2024).*

When left untreated, leprosy led to visible deformities, including claw hands, lost fingers and toes, and facial deformities. Almost all people affected with leprosy and who participated in the study experienced one or more deformities and disfigurement/s. One of the female FGD participants explained her experience by saying, *“At the beginning this disease began by forming blister on my fingers. Then it progressed to make my hands feel weak and numb.” (A 45 years old female, FGD 5, P3, 2024).*

#### Perceived cause and modes of transmission.

A predominant perceived cause of leprosy is supernatural causes, including divine punishment, curses, or evil spirits. Due to these perceptions, individuals delayed seeking timely medical treatment and seeking spiritual or traditional remedies instead. Some respondents attributed their condition to God’s will, framing it as a test or unavoidable fate. One FGD discussant explained this as: *“I believe I had this disease because God allowed me to have it. It is already stated in both Hadith and the Quran that every person carries this disease in their heart, but only those permitted by the supernatural to show its signs and symptoms become like us” (A 45 years old male, FGD 4, P4, 2024).*

#### Misconceptions and awareness.

There is a deep-rooted misconception about leprosy in the community, particularly in rural settings. As a result, in many individuals, the disease progressed as they sought religious or traditional remedies. One FGD discussant explained as: *“As my parents did not know what disease I had at the beginning, they considered as if I had the disease due to allergy. They gave me a lot of traditional medicine. I drank and ate like meat of inedible animals* ***(foon xaddee faa na naachisu turan.)*** *(A 65 years old male, FGD 6, P6, 2024).*

Many of the participants believed that leprosy is a non-infectious condition. One informant described this as: *“…, Some people say it is from a germ. However, this disease is not germ. It is a gift from God* ***(kennaa rabbiiti)****. Angels brought it from God and transmit it to human beings” (A 60 years old male, KII 5, 2024).*

Another informant explained this misconception as:

*“When I was young, there was an elderly woman with leprosy who had lost her hands and legs, and eventually, she passed away. After her death, the local community became very afraid. They believed the disease could be passed on through her clothes and belongings. Because of this fear, they let her house fall into ruins and were even too scared to burn it or her possessions, thinking the disease might spread through the air****. “In local language she said: Mana dubartii sanaa gubuuf jedhanii, yoo gubne aarri manichaa dhukkuba juuzaamaa nutti fida jedhanii gubuu dhiisan.”*** *(55 years old female, FGD 6, P3).*

### Social and psychological impacts

#### Social impacts.

There is stigma and discrimination towards people affected by leprosy remains a significant challenge. Many individuals affected by leprosy face a derogatory word that is linked to leprosy-associated stigma and discrimination. One FGD discussant explained as: “*Our community calls individuals affected by this disease as* ***qomaaxaa, qurcii*** *and* ***Juzaamaa****” (A 60 years old male, KII 5, 2024).*

Stigma and discrimination have detrimental impacts on the emotions of the people. Many individuals affected by leprosy lack hope for life. Even though stigma from people without a history of leprosy is apparent to those affected by leprosy, there is also stigma from within affected individuals themselves, based on the presence or absence of disabilities. One informant explained this as:

*“For example, when there is wedding ceremony in the house of the people who had been leprosy patient and get healed from the disease without having any scar on their body, they do not discriminate us.” (A 55 years old female, FGD6, P3, 2024).*

Individuals affected by leprosy face rejections at schools. One key informant explained this as: *“Even, recently I went to school for some issue and I kept my letter of the request on the table of the chair person. Then the school director kept my paper away by pushing it with pen she had at hand. She refrained from touching my letter of request with her hands” (A 70 years old male, FGD5, P1, 2024)*

#### Inter-generational stigma.

There is a deeply rooted inter-generational stigma associated with leprosy, which affects children of leprosy-affected families. As a result, children of leprosy-affected individuals face social exclusion, bullying, low self-image, and self-imposed restrictions at schools and other social gatherings. A female key informant explained this as:

*“Children of people affected by leprosy began to form friendships with children from families with no history of the disease. One day, there was a wedding at the home of those without leprosy. On such occasions, people affected by leprosy and their children were often abandoned, even though they had friendships outside the home.” (A 60 years old male, FGD 6, P2, 2024).*

*Another informant explained bullying words at school as:*

*“…there is a difficult problem in school. In some schools, they call our children by saying “Yekokor lijoch”; in Addis Ababa, they call ‘Ye Zingible lijoch’[to mean ginger’s family] due to the shortening and absence of fingers on our hands.” (A 50 years old female, FGD7, P7, 2024)*

#### Self-stigma and concealment.

Many individuals affected by leprosy conceal their condition due to self-stigma. A key informant explained this as: *“I completed my treatment without informing my family members (children and wife) and close relatives about my condition”* (A 18 years old female, KII 01, 2024). The cycle of stigma starts with delayed care because of self-stigma and perceived stigma. This delay leads to disabilities and visible disfigurement. It triggers social rejection, disrupts treatment, and forces people to relocate to escape discrimination. As a result, the disease progresses and continues to spread. Stigma grows stronger, leading to exclusion, family betrayal, and isolation. Psychological distress, including hopelessness and suicidal thoughts, also emerges. Fear of stigma then prevents others from seeking care, and healthcare workers’ neglect keeps the cycle going.

#### Psychological effects of leprosy.

Many people affected by leprosy face significant psychological challenges. The psychological impacts of leprosy also impact their children. A female key informant explained this as:

*“…. Our children get into fights with others who insult them and often find themselves in complicated situations. For me, if anyone insults me, it means nothing. Why? Because I have the disease and have accepted the challenges associated with it. But for our children, this ends with a severe psychological trauma.” (45 years old male, KII 3, 2024).*

Some individuals fall into severe psychological distress and commit suicide or pray to die due to the stigma they faced in the community. One FGD discussant explained this issue as: *“One day, I got angry and went to drink* ***‘teji’*** *[local alcoholic beverage], hoping it would kill me. I thought drinking* ***‘teji’*** *could end my life, but it didn’t.” (A 72 years old female, FGD6, P7, 2024).*

#### Impact on married life.

Leprosy has profound impacts on the marital lives of individuals affected by the disease. As a result, many individuals have marriage arrangements among the leprosy-affected individuals. Often, children of leprosy non-affected families reject marriage proposals from leprosy-affected individuals due to fear of being insulted. A male key informant explained this as: *“We prefer to arrange marriages within our community to avoid the stigma and discrimination associated with marrying outside of our community” (A 38 years old female, FGD 7, P3, 2024).*

If marriage is not arranged through the marriage arrangement approach, many individuals prefer to stay unmarried. A female key informant explained this as:

“*Many of our children here choose to remain unmarried due to the fear of insult because of their family’s conditions. They worry about being disconnected from their families and fear their spouse may not accept them or might refuse to visit their family during holidays or funerals. They say*, “I know your family; they don’t even have hands to eat food from a dish.”. ***Isaan warra biddena kutatanii hin nyaannedha.****” (A 32 years old female, KII 13, 2024).*

Marriage, which happens between children of leprosy-affected and those from leprosy-free families are assumed to be more likely to end in divorce. One FGD discussant explained this as: *“Let alone forming new marriages with someone who has leprosy, many healthy husbands whose wives contract this disease end up divorcing them because of it” (A 38 years old female, FGD 7, P3, 2024).*

Another FGD discussant added even: “*…, husband of my sister divorced her because of my conditions. Before, my sister had children with her husband. However, when he saw me developing severe disabilities due to leprosy, he divorced her.*” (*A 70 years old male, FGD 5, P1, 2024).*

Leprosy-affected individuals are seen as a threat to their siblings and may be rejected by their parents. Most families force their children who are affected by leprosy to change address. A female informant explained this as: *“My family told me to go somewhere else so that my siblings could get married. They fear the fate of my brothers and sisters will not be good if I stayed with them.” (A 42 years old male, FGD 01, P2,*2024).

### Economic and educational challenges

#### Economic impacts.

Leprosy-affected individuals often face significant economic hardships resulting from systematic rejection of those affected by leprosy in the recruitment process, even in labor activities. One key informant explained as:

*“…., Even government organizations do not consider people with leprosy when they seek to hire human manpower for some activities. They exclude us by the criteria. Most of the time physical fitness is considered a criterion. They say in Amharic, “Tenenitu yetemola” when they post vacancy. So, anyone with disabilities doesn’t get job due to those criteria. Rejection by criteria” (A 60 years old male, KII 2, 2024).*

Due to the economic impact, access to essential medications for managing leprosy is limited, as the patient could not afford the additional drugs needed to treat the side effects of MDT. As a result, many individuals were forced to discontinue even MDT and later end up with amputations. A female informant explained this as: *“The medication used to treat leprosy is free of charge, but there are additional medications prescribed. They said it is crucial for the management of the side effects of leprosy treatment. However, I couldn’t manage to buy the drugs due to a lack of money (IDI, 2024).*

#### Impact on educational opportunities.

Leprosy has a profound impact on educational opportunities. While few students can withstand this insult and complete their education, many others are forced to interrupt their education due to bullying and gossip at school.

A FGD discussant explained this issue as: *“Regarding the lack of educational opportunity, I had this disease when I was in grade two. Because of the disease, I had to discontinue my schooling. Most of my classmates are now able to complete schooling and are government employees. As a result, I struggle with daily life and earning income* (FGD, 2024).

Another participant described his experience of persistence and being able to complete schooling as:

*“…They [children from leprosy families] drop out because other students insult them, calling them ‘qurcii, qurcii.’ [derogatory term to describe someone with disfigurement due to leprosy]. Some students leave school because they can’t stand this insult. I have faced these challenges. Students make fun of us, saying they come from leprosy families. However, I have withstood all those insults and today am working as a teacher” (IDI, 2024).*

Due to economic challenges, many children stop school because of a lack of food and other educational materials. A FGD discussant explained this as: *“Most children drop out of school due to the lack of adequate food. They end up scavenging food in a dustbin or doing other similar activities to earn a little income. If we had enough food for our children, they would succeed in their studies just like other children,*” (FGD, 2024).

### Vulnerability (women and children)

The study participants emphasized that women and children bear a disproportionately higher burden of leprosy, due to their immune status and social roles. A female key informant described this as: *“Leprosy is a greater burden for women and children. Because women work in the kitchen, mostly using fire, they are exposed to fire when cooking food for their family and may sustain unnoticed injuries due to loss of sensation. When we come to children, their immune systems are not as strong as that of adults.” (IDI, 2024).*

### Leprosy management and healthcare challenges

#### Preference for traditional and religious remedies.

Many individuals prefer traditional and spiritual remedies to medical care for leprosy. One female affected by leprosy explained:

*“I did not want a health facility following the initial symptom of leprosy. I rather sought out religious healing places, such as holy water and prayer areas. Finally, when I realized that all of these remedies did not work, I went to a health facility and received treatment. Unfortunately, most of my fingers were now resorbed.” (IDI, 2024).*

#### Treatment interruption.

Lack of family support is also leading to treatment interruptions, particularly in children. One FGD discussant described her experiences as: *“…then I went to the health facility to seek care, and they gave me drugs. But since I was a child, I have not been taking the medication correctly. As a result, the disease worsened, and my hands and legs developed wounds.****”*** *(FGD, 2024).*

Another participant added: *“…, but since I was a child, around 10 years old, I dumped the medication in the soil and did not swallow it. Had anyone supported me during that time, I could have saved my fingers” (FGD, 2024).*

#### Systemic complications from untreated leprosy.

Leprosy-affected individuals often discontinue treatment and return to religious practices like “tsebel” (holy water) for leprosy management. Discontinuation of medication was a common problem, often leading to worsening disease progression such as severe ulcers and deformities. Untreated leprosy causes loss of sensation, leading to ulcers on the hands and legs during work, which can deteriorate over time and become life-threatening. One female discussant described this as: “When *individuals affected by leprosy go untreated, ulcers form on their hands and legs. These ulcers progressively worsen and ultimately lead to death. These ulcers occur when these individuals work. This is due to their loss of sensation. “ (IDI, 2024).*

Another participant added: *“Due to our late health care seeking, our body became handicapped. If we had sought health care on time, we could have saved our body parts.” (IDI, 2024).*

Accessing appropriate healthcare is challenging, particularly for those who have developed disabilities. One in-depth discussant described her life journey as:

*“I have suffered from my leg wound for the last 30 years. It improved when I received treatment, but later relapsed. Now, doctors told me it has affected my bone, and I need to be treated in Addis Ababa, as the physician who can perform the procedure is not available in this hospital. Although hospitals that can provide corrective surgery are available in this city, I cannot afford their cost.” (IDI, 2024).*

#### Denial of care at the health facility.

Denial of care and support at the health facility is another challenge faced by individuals receiving treatment for leprosy management. Upon return to the health facility, they are often mistreated by healthcare workers. A key informant explained these issues as:

*“Nearly 30 years ago, when I began treatment for leprosy, thieves stole my remaining tablets before I complete it. I reported to the hospital and requested for refill. However, the health care worker at that facility refused to replace my medication, citing that it was my fault. He spoke harshly and treated me without compassion. Without receiving a refill of the lost tablets, I returned to my village and left my fate to God. Unfortunately, my condition relapsed and worsened over time. I ended up in the hospital”* (KII, 2024)

*Another female key informant shared her experiences as:*

*“For instance, when I went to the dentist to remove my painful tooth, I got the medical registration number, but the HCWs were not willing to provide me the service I demanded. I was thinking that, hadn’t been disabled, I believe they would have gladly provided the service like others… My child, witnessing the situation, advised me to leave immediately and promised to help me find another clinic for my tooth extraction” (IDI, 2024).*

The repeated refusal by multiple healthcare providers to physically examine the patient’s affected body parts reflects a pervasive fear and negative attitude towards the disease. Another informant added:

*“The health care providers are not happy touching our bodies. I don’t want to recall the painful moment I experienced. I went to a health facility and waited in line. When I got into the examination room, the first doctor refused to examine me and called someone else in. This new provider looked at my affected body part and quickly left to get another healthcare worker. Throughout this process, I only saw one worker by the end. I felt frustrated. The last health care worker seemed to examine my body parts without care or compassion, only to meet his duty. He prescribed medication without even talking to me, then left to wash his hands. After that, I saw him washing his hands with soap again and again. I remember he had a look of hate and discrimination on his face.” (FGD, 2024).*

Health facilities lack a welcoming climate for leprosy-affected Individuals. Many affected individuals are not happy with the care and support they received at health facilities. One FGD discussant described this as: *“Even if an affected person visits a health facility, there is no adequate medication, and the health facilities are not as welcoming as they used to be.” (Female FGD, 2024).* Another informant added: *“There is a knowledge gap among healthcare providers in diagnosing and treating leprosy.” (IDI, 2024).*

*Another informant explained as:*

*“…, Recently, I injured my hand and went to hospital. The doctor examined me properly and gave me a prescription to get medication from a private pharmacy, saying that the hospital didn’t have it. When I got to the pharmacy, the pharmacist took my prescription, paused for a moment, and asked my name again. I told him my name, and he threw the prescription paper at me. I cried a lot.” (A 60 year old female, FGD 7, P4, 2024)*

### Community and stakeholder involvement

#### Stakeholders involved in leprosy management.

The study participants explained the diminishing number of stakeholders engaged in leprosy management. One of the most notable stakeholders from the earliest time to today is the German Leprosy and TB Relief Association (GLRA). GLRA is not only involved in medical care but also in humanitarian support. A FGD discussant explained the engagement of GLRA in leprosy as:

*“It was GLRA that has been supporting us in this rehabilitation center. They provide us with about eight or nine kilograms of teff powder, legumes, pepper, and soap every month. They are also supported by constructing a house and providing some livestock.” (FGD, 2024).*

Other stakeholders such as The Leprosy Mission International, the Ethiopian Red Cross Society, the Oromia Social Affairs Office, the Ethiopian Association of people affected by leprosy, Jimma-Bonga Catholic Organization, and other charitable organizations. These organizations often engaged in awareness raising about leprosy, providing educational support for children, facilitating a credit service, and providing skill training.

One participant shared his experiences as: *“The organization that supports us is a Catholic organization. I would like to sincerely acknowledge them. Jimma-Bonga Catholic has been supporting us. The social service office also supported us.” (IDI, 2024).*

Another informant described the support he received as:

*“I lived this way until I turned 15 years old, at which point I fled to my uncle’s house here in Addis. He brought me here and left me. I then completed my medication and am now living in a house built for people discharged from the hospital who have no family or support. I know my family would not accept me if I went back*.” (FGD, 2024).

However, participants consistently raised concerns regarding the adequacy and consistency of the support they received. Several noted that the support has diminished over time and remains insufficient in addressing the actual need. One informant explained this as: *“The aid given is not enough compared to the number of people affected and the severity of their condition?” (FGD, 2024).*

In addition to diminishing support, the informants highlighted a significant governance gap marked by unclear institutional responsibility. One informant explained this by questioning as: *“…, it is not even known who has to deal with the leprosy: the association, the health ministry or social affairs?” (IDI, 2024).*

#### Presence of undiagnosed cases and absence of active case screening.

One of the most pressing issues is the continued presence of individuals affected by leprosy who remain hidden at home due to fear of stigma and discrimination. This problem emanates from misconceptions about the disease and lack of awareness. A female informant explained as: “*Even now, there are a lot of people suffering from this disease, staying at home.” (*Female FGD, 2024).

### Coping mechanisms and resilience

#### Treatment efficacy and the importance of timely care.

The study participants confessed effectiveness of leprosy treatment if the affected individuals seek care immediately after the initial symptoms. Early diagnosis and prompt initiation of MDT are essential for preventing the development of disabilities associated with leprosy.

A female discussant explained as: *“If individuals affected by leprosy seek care immediately after the initial symptom of leprosy, such as white skin patches at a health facility, it can be cured without developing any disabilities.” (IDI, 2024).* Another discussant added as: “…., *thanks to God, due to my commitment and determination to seek healthcare at Alert Hospital, today I can at least eat food with my hands. It means I saved my hands.” (IDI, 2024)*

#### Resilience.

Individuals affected by leprosy established their social connections. This includes marriage arrangement within their own community in response to the stigma and discrimination they face from the broader society. This led to the establishment of an own Idir of the community of leprosy affected individuals (Idir is a term in the local language which, in Ethiopia, refers to a traditional community-based mutual support association that can be translated as a ‘self-help group’ or ‘mutual aid association) to support each other economically and socially. One of the participants stated this as:

*“Concerning idir, in our idir there is a member who has leprosy and who does not have leprosy. But most of our idir members are the family of this leprosy association. The idir of the people who do not have leprosy may accept us as their members. But since in idir there are responsibilities of doing different tasks that we can’t equally perform with other people, we don’t go to their idir.*” *(FGD, 2024).*

Another participant added, stating: *“Now in this leprosy association, we don’t fear each other; we don’t stigmatize and discriminate against each other. We live with full happiness.”* (Female FGD, 2024) *“Despite being unable to engage in labor like others in the community, individuals* *affected by leprosy still attend funerals and weddings and participate in different aspects of social life here. We participate in various social activities, without fear of stigma.”* (IDI, 2024).

Despite this, there are positive stories where individuals from leprosy-free families accept marriage with children of leprosy-affected families. One FGD discussant shared her experience as: *“My daughter married a man whose family has no history of leprosy.”* (FGD, 2024)

Another discussant added *as: “…, for example, my daughter, who was born and raised here in Shashemene, got married to a husband who does not have the same history in his family. That is something for which I feel proud.” (FGD, 2024).*

### Recommendations for leprosy elimination

#### Enhancing awareness and education.

Community awareness and education are important to address misinformation. One mixed FGD discussant explained this as: *“…, therefore, the government should focus on raising awareness for both urban and rural communities through radio and television programs, particularly emphasizing the importance of seeking timely healthcare to reduce the risk of developing disability.” (FGD, 2024).*

Another participant also added: *“Many of us have suffered amputations due to a lack of awareness about available treatments and delays in seeking care. However, the new generation should have to aware of the importance of timely healthcare for leprosy.” (FGD, 2024).*

#### Improving healthcare access and service quality.

Health system strengthening with a focus on healthcare workers capacity building and improving supply could support efforts towards eliminations. One informant explained this as: *“I think that unless HCWs are trained well, sufficient medication and treatment are made available, leprosy cannot be eliminated from this country.” (IDI, 2024).*

Another informant added: *“Capacity-building training should be given to healthcare providers on leprosy to effectively diagnose and treat leprosy.” (IDI, 2024).* Leprosy rehabilitation centers should be strengthened. One participant explained this as: *“There should be strong rehabilitation centers for providing holistic support for those who have already developed disabilities.” (IDI, 2024).*

Comprehensive support, including psychological care, financial assistance, and provision of assistive devices, and continuum follow-up regarding the treatment, is crucial for ensuring treatment adherence. One of the informants explained this as:

*“To eliminate this disease, healthcare professionals need to monitor the progress of leprosy patients and ensure medication adherence. It is more effective if there is phone communication, allowing patients to check on the availability of medications and their adherence. They should encourage patients to take their medication properly to eliminate this disease.” (FGD, 2024).*

#### Engagement of key stakeholders and community leaders.

Engaging key stakeholders such as religious leaders, health professionals, and individuals affected by leprosy was highlighted as a strategic approach to combat leprosy-associated stigma and promote early care-seeking. One informant explained this as: *“Every person in the community, government body, and NGOs should have to play their role to eliminate this disease.” (FGD, 2024).*

Another key informant added:

*“Religious leaders, according to their respective religions, should play their roles in educating their followers about the importance of seeking healthcare when they notice symptoms of leprosy. Furthermore, it is their responsibility to promote the reduction of stigma and discrimination associated with leprosy.” (FGD, 2024).*

Another participant highlighted the importance of involving individuals affected by leprosy, as:

*“We, those affected by leprosy, can do many things. We are competent and educated. We have a healthy brain. Only our hands and legs have been affected. With support, we could accomplish much through our voices. For instance, I wish to be like birds, flying over the sky and informing everyone that leprosy is curable with medications. No one deserves this suffering afterward.” (FGD, 2024).*

## Discussion

Stigma and misconceptions remain obstacle to leprosy management impacting the health, social, and financial aspects of the affected individuals. Many individuals still view leprosy as a hereditary condition, a curse, a punishment from God, or something caused by other supernatural forces. Some also believe they were chosen by God to suffer on earth to gain salvation. These views likely stemmed from historical misconceptions, cultural beliefs, poor health practices, and a lack of awareness.

Misinformation, delayed diagnosis and treatment, physical disfigurement, and social rejection are the main causes of the stigma. This stigma is exacerbated by the ensuing disabilities, which worsen patients’ suffering and aid in the ongoing community spread of leprosy. Fear of discrimination and shame discourage people from seeking care. Stigma towards leprosy-affected individuals emerges from multiple sources. This includes self-stigma, stigma from the general community and healthcare workers, and stigma from individuals affected by leprosy who have not developed observable signs.

Study participants also explained the presence of hidden community cases. As a result, many individuals affected by leprosy remain undiagnosed and hidden within their communities due to persistent stigma, fear of discrimination, and limited awareness about the disease. This problem is coupled with a lack of active case finding strategies and community outreach leading to delayed diagnosis, continued transmission, and increased risk of disability among the affected individuals. The findings imply that stigma, fear, and lack of awareness continue to hinder early detection of leprosy, leaving many cases undiagnosed and untreated.

Other studies also confirmed that a considerable proportion of individuals with leprosy remain undiagnosed and untreated within the community undermining the global leprosy data. A survey in Ethiopia [5], Bangladesh [17] and India [18], confirmed the presence of numerous untreated leprosy cases. The presence of undetected infections presents a major obstacle to the disease elimination efforts by increasing the overall disease burden and sustaining its transmission.

Leprosy has huge economic impacts for all affected persons, perpetuating a cycle of poverty by pushing the affected individuals and their children from education and employment opportunities. Next to the economic effects, social rejection has a negative impact not only on leprosy patients but also on their families at large. Children whose families are affected by leprosy are humiliated and labeled with derogatory names such as ***“the leper family,” “dogmani,” and “ginger’s family,”****, “****Yekuteba lijoch”*** which increase their isolation from the larger society. In order to protect family members, many leprosy affected individuals are forced to quit their treatments and relocate to another place. Furthermore, this impacts multiple aspects of social life such as marriage. This indicate that leprosy makes the affected individual more vulnerable to other non-communicable diseases, such as mental health disorders, diabetes mellitus, and hypertension [19,20].

Studies from other global leprosy-endemic regions have shown comparable findings about stigma. For instance, leprosy-affected individuals face social exclusion and verbal abuse to the extent that they feel ***“dead while alive”*** and hide their diagnosis to avoid shame in India [21]. Similarly, Individuals affected by leprosy are excluded from education, marriage, and religious activities, even after being cured in Nepal [22]. These highlight the global nature of leprosy-related stigma and underscore the need for comprehensive, culturally sensitive interventions to address its deep social and psychological consequences.

Leprosy affected individuals endure bullying, denial of services, and social rejections, hampering the ongoing global efforts to end leprosy. As a result, they tend to pursue traditional and religious remedies instead of medical treatment. These findings were in line with historical and theological views of leprosy, where leprosy has been imbued with symbolic and ritual significance [23].

**Fig 2:**
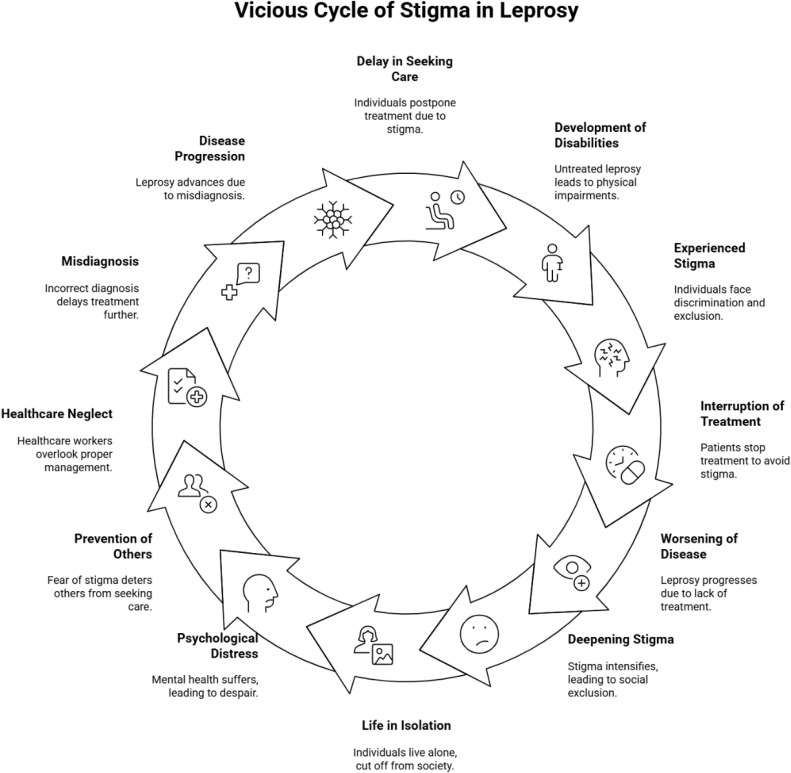
Vicious cycle of stigma and Its consequences (created using Napkin) [16].

The stigma around leprosy creates a harmful viscous cycle (stigma → concealment → delayed care → disability → more stigma) that affects individuals in medical, social, and economic ways. Leprosy affected individuals frequently conceal their illness, isolate themselves from others, and some eventually resort to suicide attempts (Fig 2).

Even though leprosy stigma has its own culture and structures in endemic settings, including India, Brazil, and Southeast Asia, it again and again follows typical patterns of exclusion and delayed care. In India, moral stigma and gendered inequalities are perpetuated through beliefs that link leprosy and karma or God’s wrath [21]. Racialized and structural stigma is perpetuated through colonial legacies, as observed in Brazil [24]. Nepal and Nigeria stigma still hamper treatment and patient rehabilitation due to religious ideologies [25,26].

Leprosy remains one of the most neglected diseases, even within the broader category of neglected tropical diseases. This persistent neglect has contributed to the endurance of antiquated beliefs and deeply rooted social and spiritual myths surrounding the illness. Over the past several decades, there has been limited engagement and deliberation among stakeholders through scientific workshops or professional forums, despite the significant sequelae associated with leprosy. As a result, leprosy receives minimal attention in the national health system, academia, research & development and from funders perspectives.

Consequently, the stigma and social neglect linked to leprosy have caused greater suffering and marginalization than the biological effect of the disease. Neglect of leprosy by the health system has profound consequences. Frontline healthcare workers often lack adequate exposure and training, leading to misdiagnosis, increased leprosy-related disability, and ongoing disease transmission. From a public health standpoint, delayed management of infectious diseases facilitates transmission to healthy individuals, amplifies community-level spread, and imposes a substantial burden on the healthcare system. Leprosy remains one of the most neglected diseases in Ethiopia. This statement is corroborated by the circumstance that the country is even absent from the list of nations that have recently updated their leprosy control strategies, indicating limited attention by policy makers.

In societies with long-standing misconceptions about leprosy, also health care workers can show stigmatizing behavior towards patients. Here, health services with their HCWs will not be seen as an environment of confidence and trust, which is an indispensable prerequisite for the management of an already stigmatized infectious disease.

Regardless of a patient’s condition, healthcare professionals have a moral and professional obligation to treat them with fairness, dignity, and respect. The World Medical Association (WMA) International Code of Medical Ethics (2022) [27] states that doctors must treat patients with competence, promptness, and compassion; practice medicine equitably and without discrimination; respect patients’ rights and autonomy; and prioritize their health and well-being while causing the least amount of harm.

The basic human right of individuals affected by leprosy are at stake. The Alma-Ata Declaration from 1978 recognized health as a basic human right and supported Primary Health Care (PHC) with an emphasis on fairness, community involvement, and dealing with social factors that affect health. However, individuals affected by leprosy feel and are left behind by global health programs. This goes against the aims of initiatives such as the Universal Health Coverage (UHC) [28] and the Sustainable Development Goals (SDGs) [29], which strive to guarantee health and well-being for everyone.

The dilemma has also been formulated by the United Nations Human Rights Council Resolution 29/5 (2015) [30], which highlights that the ongoing discrimination against individuals affected by leprosy is a severe violation of their rights to dignity, equality, and full participation in society [30,31]. The results of this study also depict that leprosy patients’ rights are neglected or violated. The Patient’s Charter for Leprosy Care affirms the rights of affected individuals to respectful care, non-discrimination, and involvement in decisions about their treatments. Although there are effective antibiotics for the treatment of leprosy, the patients often report isolation, verbal mishandling and exclusion in the context of their health care systems which are clear violations of the Charter’s essential principles [32].

There seems to be a gap between health policy and practice on the ground, due to the absence of clear, actionable implementation of policies. Leprosy management should extend beyond medical care to include the social and psychological dimensions of the disease. To accelerate progress toward a leprosy-free world, it is essential to place greater emphasis on research and development. Moreover, healthcare for leprosy-affected individuals must be provided in fair and respectful ways, with sympathy and more focus on protecting their rights and dignity [29].

Despite the bio-psycho-socioeconomic impacts, some individuals affected by leprosy can develop resilience by creating their own social support systems comprising social groups and communal living arrangements with minimal stigma. There were individuals who sought early treatment and able to avert leprosy related disability. There were also individuals who were able to create social connections like marriage life of their children with those from leprosy free families. It appears that community mobilization, through creating awareness, providing compassionate healthcare, and giving appropriate support can break the cycle of stigma and social exclusion. Several community-based initiatives have shown to be successful in reducing the stigma attached to leprosy. These included education and peer support, advocacy and experience sharing, rights-based and cognitive-behavioral therapy, and community engagement programs like PLA (Participatory Learning and Action) and STEP (Social Transformation and Empowerment Process), which helped identify local causes of stigma and develop inclusive, context-specific solutions. Additionally, media campaigns and community sensitization via theatre, radio, or storytelling, training medical professionals and community leaders, and involving leprosy-affected people as advocates or trainers all helped to lessen stigma [33–36].

### Limitations of the study

One of the limitations of this study is its cross-sectional design and the lack of a stigma-reduction intervention. We would like to suggest longitudinal follow-up studies that would be able to assess the effects of stigma reduction interventions on care-seeking behavior over time. In addition, there was a potential selection bias from sampling patients at specified leprosy care facilities and the study’s concentration on hotspot regions. This may limit the external validity of our findings. Because neither the general public nor medical professionals were involved, we can only provide insight into the perspectives of leprosy affected individuals. Furthermore, participants might have underreported experiences of stigma or over-reported socially acceptable opinions due to social desirability bias.

## Conclusions

This study shows that leprosy remains a major public health issue in Ethiopia. Although the introduction MDT has brought a paradigm shift in leprosy management, the existence of such misconceptions continues to pose a barrier to the effective utilization of MDT. Its impacts go beyond physical disability, encompassing social, psychological, and economic consequences. Misunderstandings about the disease, reliance on religious and traditional remedies, and delays in seeking care perpetuate development of visible organ damage and its ongoing transmission. Health system challenges, such as limited skills in managing leprosy at the primary care level and refusal of care by some healthcare workers, worsen the situation. To accelerate leprosy elimination, the social, psychological, and economic dimensions of the disease must be addressed in addition to medical management. Additionally, individuals affected by leprosy should be empowered to participate in decision-making and advocate for themselves. Moreover, leprosy stigma-reduction and care interventions should be incorporated into Ethiopia’s primary healthcare system in order to improve accessibility, sustainability, and effective disease control. Eventually, we must conclude, that Ethiopia is still far from the “Towards Zero Leprosy, 2030” roadmap.

## Supporting information

S1 File Codebook. (DOCX)

S2 File Glossary of terminologies. (DOCX)
