# Supplementary material for: Exploring the health-seeking journeys of individuals affected by leprosy: Lived experiences in selected urban rehabilitation centers in Ethiopia
Source: PLoS Negl Trop Dis. 2026 Feb 17;20(2):e0013938. doi: 10.1371/journal.pntd.0013938 (PMC12931759; doi:10.1371/journal.pntd.0013938)
Supplement: S1 File — (DOCX) [file pntd.0013938.s001.docx]

Codebook

Code System

| 1 Stakeholders involved | 30 |
| --- | --- |
| 1.1 Organizations involved in leprosy management | 0 |
| 1.1.1 Association people affacted by leprosy | 21 |
| 2 Lack of education opportunity | 20 |
| 3 Distorted Marriage life | 40 |
| 3.1 Concerns for children and siblings marriage life | 7 |
| 3.2 Divorce | 10 |
| 4 Economic Impacts | 75 |
| 4.1 Job Application Rejection | 19 |
| 4.1.1 Youth marginalization | 1 |
| 5 Disability | 39 |
| 5.1 Ambutations | 5 |
| 6 Suicide | 6 |
| 7 Regret | 14 |
| 8 Social impacts | 55 |
| 8.1 Disconnection from society | 13 |
| 9 Systemic complications | 38 |
| 10 Psychological impacts | 25 |
| 11 Non-discrimination | 43 |
| 12 Positive HC experiences | 48 |
| 12.1 Treatment efficacy | 68 |
| 13 Stigma | 164 |
| 13.1 Inter-generation stigma | 12 |
| 13.2 Experienced stigma | 113 |
| 13.2.1 Labeling and Discrimination | 73 |
| 13.2.2 stigma by HCWs or at HFs | 14 |
| 13.3 Perceived stigma | 13 |
| 13.4 Self stigma | 53 |
| 14 Barriers to care and management | 100 |
| 14.1 Patienst or parental | 0 |
| 14.1.1 Parental medicalcare abandonment | 19 |
| 14.1.2 Delay in treatment | 60 |
| 14.1.3 Perception about leprosy | 61 |
| 14.1.3.1 Awerness on leprosy | 49 |
| 14.1.3.1.1 Healthcare services in leprosy | 37 |
| 14.1.3.2 Perceived cause of leprosy | 79 |
| 14.1.3.3 Perceved mode of transimmission | 39 |
| 14.2 Cross-cutting | 0 |
| 14.2.1 Relapse | 7 |
| 14.2.2 Treatment interruption | 15 |
| 14.2.3 Language barrier | 1 |
| 14.2.4 Neglected | 54 |
| 14.2.5 Presence of active cases | 14 |
| 14.2.6 Reliance on external support | 5 |
| 14.3 Lack of services at healthcare facilities | 0 |
| 14.3.1 Lack of local capacity for leprosy | 104 |
| 14.3.2 Lack of support | 37 |
| 14.3.3 Mismanagement | 12 |
| 14.3.4 misconception about the disease | 77 |
| 15 Care seeking | 73 |
| 15.1 Seeking medical care | 18 |
| 15.2 Non healthcare faciliity | 63 |
| 16 Symptoms and Causes | 89 |
| 17 Need for support | 117 |
| 18 Vulnerability | 20 |
| 18.1 Gender aspect of leprosy | 24 |
| 19 Coping Mechanisms | 11 |
| 19.1 Social connection with shared health conditions | 76 |
| 19.1.1 Arranged Marriage | 9 |
| 19.1.2 Conflict Resolution | 5 |
| 19.2 Fight for right | 15 |
| 19.3 Resilience (ignore stigma) | 37 |
| 20 Recommendations | 109 |
| 20.1 Strengthen leprosy rehablitation centers | 6 |
| 20.2 Capacity building | 92 |
| 20.3 Active case screening | 18 |
| 20.4 Engage stakeholders | 67 |
| 20.5 Destigmatize leprosy (learn from HIV) | 11 |

1 Stakeholders involved

1.1 Stakeholders involved >> Organizations involved in leprosy management

1.1.1 Stakeholders involved >> Organizations involved in leprosy management >> Association people affected by leprosy

2 Lack of education opportunity

3 Distorted Marriage life

3.1 Distorted Marriage life >> Concerns for children and siblings’ marriage life

Worry about the impact of one's actions on siblings' marriage prospects.

3.2 Distorted Marriage life >> Divorce

Leaving one's home due to worsening personal condition.4 Economic Impacts

4.1 Economic Impacts >> Job Application Rejection

The text describes the narrator's unsuccessful attempt to apply for a guard position

4.1.1 Economic Impacts >> Job Application Rejection >> Youth marginalization

5 Disability

5.1 Disability >> Amputations

6 Suicide

7 Regret

8 Social impacts

8.1 Social impacts >> Disconnection from society

9 Systemic complications

10 Psychological impacts

11 Non-discrimination

12 Positive HC experiences

12.1 Positive HC experiences >> Treatment efficacy

13 Stigma

13.1 Stigma >> Inter-generation stigma

13.2 Stigma >> Experienced stigma

13.2.1 Stigma >> Experienced stigma >> Labeling and Discrimination

Derogatory terms used to refer to people with disabilities.

13.2.2 Stigma >> Experienced stigma >> stigma by HCWs or at HFs

13.3 Stigma >> Perceived stigma

13.4 Stigma >> Self stigma

14 Barriers to care and management

14.1 Barriers to care and management >> Patients or parental

14.1.1 Barriers to care and management >> Patients or parental >> Parental medical care abandonment

14.1.2 Barriers to care and management >> Patients or parental >> Delay in treatment

14.1.3 Barriers to care and management >> Patients or parental >> Perception about leprosy

14.1.3.1 Barriers to care and management >> Patients or parental >> Perception about leprosy >> Awareness on leprosy

14.1.3.1.1 Barriers to care and management >> Patients or parental >> Perception about leprosy >> Awerness on leprosy >> Healthcare services in leprosy

14.1.3.2 Barriers to care and management >> Patients or parental >> Perception about leprosy >> Perceived cause of leprosy

14.1.3.3 Barriers to care and management >> Patients or parental >> Perception about leprosy >> Perceved mode of transimmission

Subcodes for 'Transmission mode':

1. Familial Transmission

- Transmission through marriage into the family

- Inheritance within family lineages

2. Interpersonal Transmission

- Transmission between individuals through close contact

- Spread through social interactions

3. Environmental Transmission

- Transmission via shared spaces or objects

- Spread through exposure to contaminated surroundings

4. Vector-borne Transmission

- Transmission facilitated by an intermediate carrier

- Spread through biological vectors like insects or animals

5. Airborne Transmission

- Transmission via respiratory droplets or aerosols

- Spread through the air over short or long distances

14.2 Barriers to care and management >> Cross-cutting

14.2.1 Barriers to care and management >> Cross-cutting >> Relapse

14.2.2 Barriers to care and management >> Cross-cutting >> Treatment interruption

14.2.3 Barriers to care and management >> Cross-cutting >> Language barrier

14.2.4 Barriers to care and management >> Cross-cutting >> Neglected

14.2.5 Barriers to care and management >> Cross-cutting >> Presence of active cases

14.2.6 Barriers to care and management >> Cross-cutting >> Reliance on external support

14.3 Barriers to care and management >> Lack of services at healthcare facilities

14.3.1 Barriers to care and management >> Lack of services at healthcare facilities >> Lack of local capacity for leprosy

14.3.2 Barriers to care and management >> Lack of services at healthcare facilities >> Lack of support

14.3.3 Barriers to care and management >> Lack of services at healthcare facilities >> Mismanagement

14.3.4 Barriers to care and management >> Lack of services at healthcare facilities >> misconception about the disease

15 Care seeking

15.1 Care seeking >> Seeking medical care

15.2 Care seeking >> Non healthcare facility

16 Symptoms and Causes

17 Need for support

18 Vulnerability

18.1 Vulnerability >> Gender aspect of leprosy

19 Coping Mechanisms

Strategies employed to manage difficult circumstances or experiences.

19.1 Coping Mechanisms >> Social connection with shared health conditions

19.1.1 Coping Mechanisms >> Social connection with shared health conditions >> Arranged Marriage

The text describes an arranged marriage without prior proposal.

19.1.2 Coping Mechanisms >> Social connection with shared health conditions >> Conflict Resolution

Shift from confrontation to patience in dealing with insults.

19.2 Coping Mechanisms >> Fight for right

19.3 Coping Mechanisms >> Resilience (ignore stigma)

Despite perceived weakness, the ability to hold on and persevere.

20 Recommendations

20.1 Recommendations >> Strengthen leprosy rehabilitation centers

Location of a camp for people affected by leprosy.

20.2 Recommendations >> Capacity building

20.3 Recommendations >> Active case screening

20.4 Recommendations >> Engage stakeholders

20.5 Recommendations >> Destigmatize leprosy (learn from HIV)
