# Supplementary material for: Exploring the health-seeking journeys of individuals affected by leprosy: Lived experiences in selected urban rehabilitation centers in Ethiopia
Source: PLoS Negl Trop Dis. 2026 Feb 17;20(2):e0013938. doi: 10.1371/journal.pntd.0013938 (PMC12931759; doi:10.1371/journal.pntd.0013938)
Supplement: S2 File — (DOCX) [file pntd.0013938.s002.docx]

**Supplementary file 2: Glossary**

**Dogmani** – A culturally and spiritually rooted perception of leprosy, shaped by fixed traditional beliefs that influence health-seeking behaviors and social attitudes.

**Foon xaddee na nyaachisu turan** – They used to give me porcupine meat, assuming it to be a treatment for leprosy. This reflects traditional medicinal practices based on cultural beliefs rather than biomedical understanding.

**Hidden case -** Refers to individuals in a community who have leprosy but have not been diagnosed, reported, or treated.

**Isaan warra biddena kutatanii hin nyaannedha** – They are those who are unable to feed themselves. This expression describes individuals affected by severe physical disabilities due to leprosy.

**Juzaamaa** – An Arabic term used to refer to leprosy.

**Kennaa Rabbiiti** – An Afan Oromo expression meaning “Gift of God.” It reflects a perception among some individuals affected by leprosy that the disease is divinely bestowed.

**Kuyera -** a derogatory name previously given to Shashemene leprosy treatment and rehabilitation center which means ignored, rejected, putting aside, non-valued people.

**Lived Experience** – The personal and subjective understanding of leprosy as perceived by affected individuals, encompassing experiences of illness, treatment, stigma, and social exclusion.

**Qomaaxaa** – An Amharic derogatory term used to refer to individuals affected by leprosy.

**Qurcii** – An Afan Oromo derogatory term used to refer to individuals affected by leprosy.

**Resilience** – The capacity of individuals affected by leprosy to recover from adversity, trauma, or chronic illness, demonstrating psychological and emotional strength in adapting to challenges such as stigma, discrimination, and social exclusion.

**Teji** – A traditional Ethiopian honey wine, naturally fermented and commonly consumed during social and cultural gatherings.

**Teninatu Yetamoala** – An Amharic expression meaning “a completely healthy person.” In the context of leprosy, it denotes social or employment criteria that exclude individuals affected by the disease from job opportunities.

**Tsebel** – Holy water in the Ethiopian Orthodox Christian tradition, believed to possess healing and protective spiritual powers. It is often used by individuals affected by leprosy as part of traditional or faith-based healing practices.

**Ye Kokor Lijoch** – A derogatory term referring to children of parents affected by leprosy, who may exhibit physical deformities such as finger resorption, causing their hands to resemble “kokor,” a type of locally made biscuit.

**Yekuteba lijoch** - A derogatory term referring to children of parents affected by leprosy and been amputated. It has meaning children of amputees.

**Ye Zinjibil Lijoch** – A derogatory term referring to children of parents affected by leprosy, who may display physical deformities such as finger resorption, making their hands resemble ginger roots.
